# Understanding Tourists’ Preference for Mammal Species in Private Protected Areas: Is There a Case for Extralimital Species for Ecotourism?

**DOI:** 10.1371/journal.pone.0088192

**Published:** 2014-02-05

**Authors:** Kristine Maciejewski, Graham I. H. Kerley

**Affiliations:** Centre for African Conservation Ecology, Department of Zoology Nelson Mandela Metropolitan University, Port Elizabeth, South Africa; University of Kent, United Kingdom

## Abstract

Private Protected Areas (PPAs) often use wildlife-based ecotourism as their primary means of generating business. Achieving tourist satisfaction has become a strong driving goal in the management of many PPAs, often at the expense of biodiversity. Many extralimitral species, those which historically did not occur in an area, are stocked in PPAs with the intention of increasing ecotourism attractions. Even though the ecological and economic costs of stocking these species are high, the social benefits are not understood and little information exists globally on the ecotourism role of extralimital species. This study assessed the value of stocking extralimital species using questionnaire-based surveys and observing tourists in Shamwari Private Game Reserve in the Eastern Cape Province of South Africa. No difference was found between indigenous and extralimital species with regards to the tourists’ weighted scoring system, average amount tourists were willing to pay, total viewing time, average viewing time or the likelihood of stopping to view species when encountered on game drives. During game drives a strong preference was found for the elephant (*Loxodonta africana*), lion (*Panthera leo*), leopard (*Panthera pardus*) and cheetah (*Acynonix jubatus*). With the exception of the cheetah, these species are all members of the “big five” and are indigenous. Species availability and visibility, however, may influence the amount of time tourists spend at an animal sighting. Our analysis suggests that certain extralimital species (typically larger and charismatic species) contribute to tourist satisfaction, while particularly the smaller extralimital species add little to the game viewing experience, but add to the costs and risks of the PPAs. We recommend that extralimital species introductions for ecotourism purposes should be approached with caution with regards to the risks to the sustainability of PPAs.

## Introduction

Protected Areas (PAs) have been established with the principle goals of conserving biodiversity [1]. The conservation of biodiversity therefore relies on the sustainability of PAs which includes achieving financial security. Ecotourism has emerged as a major means of self-financing PAs [2], particularly in Private Protected Areas (PPAs) that often use wildlife-based ecotourism as their primary means of generating business [3]. Achieving tourist satisfaction has therefore become a strong driving goal in the management of many PAs [4], often at the expense of biodiversity objectives.

PPAs are largely driven to achieve successful game-viewing sightings [5]. In South Africa high numbers of charismatic species are stocked in PPAs to enhance the wildlife experience [6]. Many extralimital species, those which historically do not occur in an area, have been introduced into PPAs to increase the number of species available for viewing [7], [8], under the assumption that this would appeal to tourists [9].

Even though the social benefits are not well understood, the ecological and economic costs of introducing extralimital species have been documented [7], [10]. In 2005, surveys indicated that the reintroduction of species into the Eastern Cape Province of South Africa cost between $97,500 and $1.8 million per PPA [8]. These non-indigenous species can lead to hybridization, degradation of habitat, low survival rates and displacement of indigenous species [10], [7]. The introduction of extralimital species diminishes biodiversity [11], [8], and may therefore threaten the ecological and economic sustainability of PPAs. This is of concern, especially in developing countries such as South Africa where the tourism economy is largely reliant on its biodiversity [12].

Ecotourism operators, however, are reluctant to remove these extralimital species, as they assume that this will have detrimental impact on ecotourism [10]. Management decisions, however, are typically based on anecdotal sources and not empirical evidence [13]. It is thus important to evaluate the role of extralimital species in PPAs to understand the implications of stocking these species with regards to ecotourism and conservation.

It has been suggested that it is public preference that motivates the stocking of extralimital species [6], [10]. We therefore hypothesize that tourists having a preference for viewing these species. Previous studies have investigated tourist preferences of wildlife [18], [19], [20]. However, there is a general lack of data about tourists’ preferences in terms of indigenous versus extralimital species. In this study we investigated the role that extralimital species play in ecotourism by analysing the value tourists place on viewing different mammal species. Understanding which species tourists focus on will determine whether preferences differ between species. The value placed on biodiversity in relation to human well-being is difficult to weigh [14], as biodiversity is not measurable through market value [15]. The value can thus be classified as a ‘use’ value [16] which is associated with actual use, such as enjoyment from visiting a reserve [17].

We used ecotourism activities to measure this use value, which was expressed as the time spent by tourists viewing different species. If the hypothesis of the value of extralimital species is supported, we predict that more time would be spent viewing extralimital species than indigenous species, relative to species availability.

## Methods

### Study Area and Species

**Figure 1:**
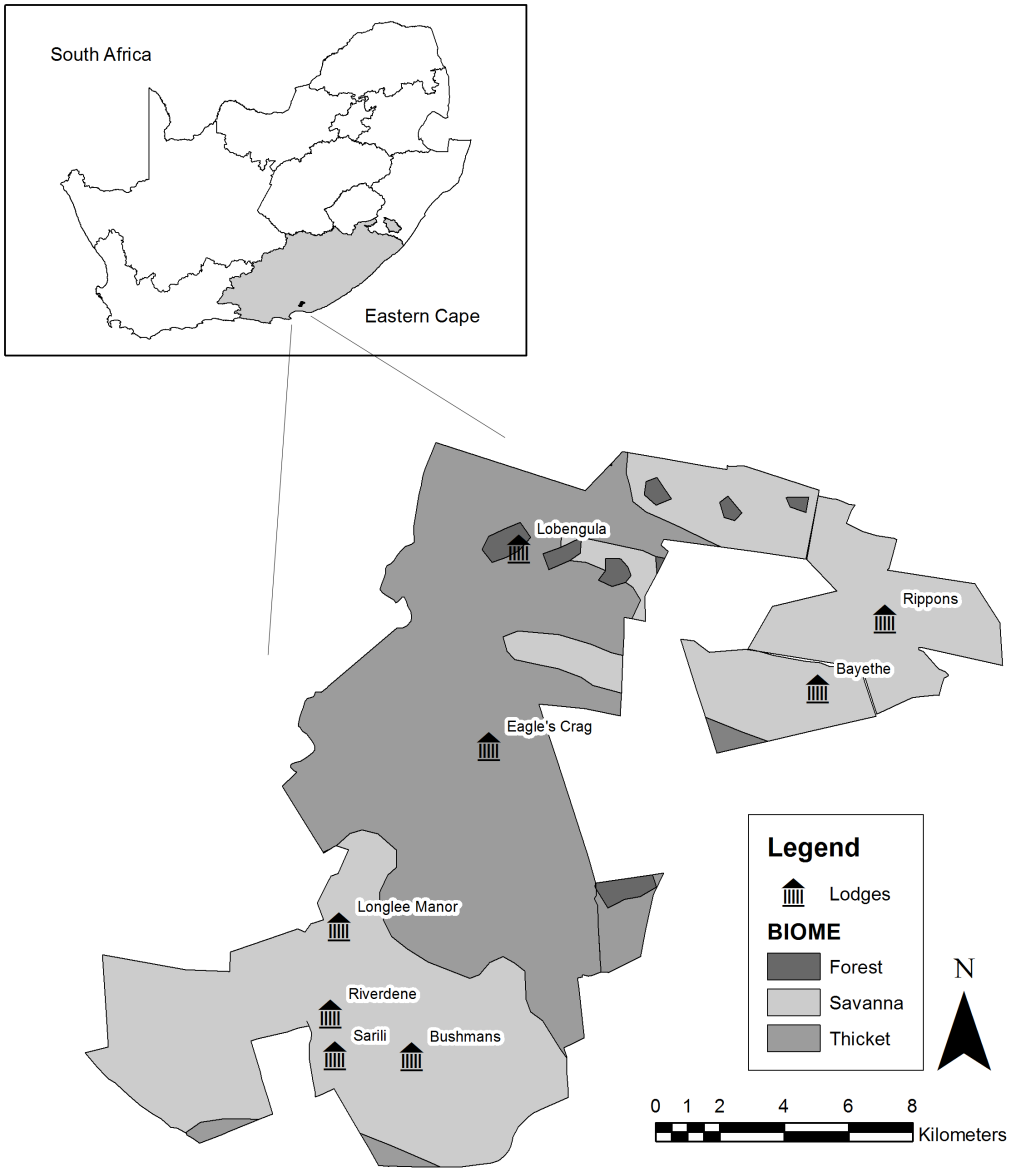
The location of Shamwari Private Game Reserve, in the Eastern Cape Province of South Africa, and the different biome types and lodges.

This study was conducted in Shamwari Private Game Reserve (between 33°20′S; 26°01′E and 33°32′S; 26°10′E) in the Eastern Cape, South Africa. Shamwari is approximately 25,000 ha in size and includes 3 of the 9 biomes found in South Africa [21] (figure 1). This reserve was used as a case study as it is recognised as an upmarket tourist destination [22]. Many national and international tourists frequent Shamwari, with an average stay of 2–3 nights [22]. Game viewing takes place through tourists being taken on an open game-viewing vehicle accompanied by an experienced guide, who also serves as the driver.

Shamwari supports a high diversity of species, including a recorded 56 mammal species. Ten of these, the cheetah (*Acinonyx jubatus*), white rhinoceros (*Ceratotherium simum*), giraffe (*Giraffa camelopardalis*), gemsbok (*Oryx gazella*), black wildebeest (*Connochaetes gnou*), waterbuck (*Kobus ellipsiprymnus*), blesbok (*Damaliscus dorcas phillipsi)*, impala (*Aepyceros melampus*), nyala (*Tragelaphus angasii*) and warthog (*Phacochoerus africanus*) are extralimital to Shamwari [23]. All members of the big five species, namely elephant (*Loxodonta africana*), lion (*Panthera leo*), leopard (*Panthera pardus*), black (*Diceros bicornis*) or white rhinoceros and buffalo (*Syncerus caffer*) are also stocked at Shamwari.

### Data Collection

Questionnaires were used to measure the tourists’ stated preference for mammal species stocked at Shamwari. The questionnaires were distributed in two different ways. Firstly, an online survey questionnaire was posted onto the Shamwari website (www.shamwari.com). This survey was posted in October 2011 and ran to January 2012. The same questionnaire was printed and given to the Shamwari guests upon arrival from October 2011 to January 2012. The first part of the questionnaire dealt with the socio-demographic information of the respondent, including country of origin, gender, age, highest educational qualification and occupation. The questionnaire then focused on the respondent’s view of Shamwari, how they heard about the reserve, whether they had previously visited other reserves in South Africa, and motivations for choosing Shamwari. A list of the mammal species found in Shamwari was presented, and the respondent was asked to rank their top five species in order of preference from 1 (most preferred) to 5 (least preferred). A weighted average was derived from these data [24] using the following equation:
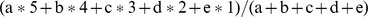



Where a = 1 (most preferred species), and e = 5 (least preferred species). In the questionnaires the respondents were provided with a list of features suggesting possible motivations that attracted the respondent to their preferred species. The respondent indicated the importance of each feature using a ranking system where 1 = most important and 5 = not important at all. The respondent was asked whether, if the preferred animal was not available in Shamwari, but available at a reserve nearby, would they still visit Shamwari or would they have visited another reserve (see appendix).

A willingness to pay approach (WTP), a contingent evaluation method [15], [16], was used to determine the value that tourists place on different large mammal species. This method is effective in making decisions and estimating monetary values for goods and services which normally don’t have prices or where no market for them exists [16], [25], [26]. The respondents were provided with a list of values and asked to select the amount they were willing to pay to see their preferred or favourite animal. A revealed preference method was not used, as we did not have the necessary data for that. In the opening statement of this question, respondents were informed that the study was carried out for academic purposes only. This was done to avoid possible bias, which would occur if the respondents believed their answers would influence pricing of the reserve. However, announcing *a priori* that the survey was an academic exercise may create ‘hypothetical biases’, an overestimation of WTP in contingent markets compared to actual payments [27], [28]. The actual WTP values were therefore not analysed, but merely ranked to compare species. The questionnaire was pre-tested among tourists to ensure it was plausible and understandable. To test for significant differences in tourists’ WTP values between indigenous and extralimital species, a two-tailed t-test was used [29]. All statistical analyses were performed in Statistica 10 (Statsoft, Inc., USA), where significance was determined at the level *p*<0.05.

Incorporating another scientific discipline into this study such as human ecology enables a better understanding of the motives behind the WTP values [30]. We therefore conducted observational studies of tourists on game drives to analyse tourists’ viewing preferences of mammal species to determine a relative measure of interest between indigenous and extralimital species. Observation studies give an accurate reflection of the tourist’s experience in the ecotourism setting [31].

Field observations took place over a period of three months (October to December 2010) when tourists were accompanied on their morning and evening game drives. For all game drives the observer sat next to the guide. All questions directed at the guide as well as conversations among the tourists could be heard from this position. A Trimble JUNO™ SB handheld GIS receiver (PDA) was used to record the data at every viewing stop using the CyberTracker software (www.cybertracker.org). An animal viewing was only classified as such when the vehicle reached a full stop. This occurred to give tourists time to observe and photograph the animal. All species that were not stopped for within a viewing distance of 0.5 km were also noted, to determine the frequency of stopping in relation to the availability of species. The GPS co-ordinates at every animal sighting were recorded using the PDA, while a stop watch was used to record the duration of the stop. In order to avoid guide preference affecting tourist viewing, the guide was instructed to follow cues from the tourists as to how much time should be spent at each animal viewing event.

The value that tourists place on different species was estimated as the duration of viewing time and frequency that each species was stopped for. The total time spent viewing indigenous and extralimital species were calculated, and a two-tailed t-test was used to test for a significant difference. The proportion of stopping to view a species when it was sighted, was classified as the likelihood of stopping to view a species. The relationship between the likelihood of stopping to view a species and the average time spent viewing the species was assessed using a linear model where likelihood was the dependent variable and average time spent viewing the independent variable. A general linear model was used to determine whether a difference was found in the likelihood of stopping to view indigenous versus extralimital species.

The proportion of time spent viewing the animal indicated the level of interest in a particular species, and the proportion of viewing time in relation to time stopped for all species indicated interest in relation to other species. To eliminate the effect of diminishing returns from repetitive game drives, tourists were only accompanied on their first game drive when species were encountered for the first time on Shamwari. The number of times each species was encountered on all game drives represented the availability of species and the frequency of stopping at each encounter to view the animal was the usage of the species. Tourist preference was determined as the difference between the ranks of usage and availability to arrange the species in order of importance, known as the tourist importance rank [32]. A Wilcoxon Matched Pairs Test was used to test for significant differences in the tourist importance rank between indigenous and extralimital species.

The manager of Shamwari Game Reserve gave permission to conduct research on the reserve. The Nelson Mandela Metropolitan University Research Ethics Committee: Human issued a written waiver of the need of ethics approval for this research, as it fell within the stipulated ethical principles and guidelines for the protection of human subjects [33].

## Results

The response rate of the questionnaires was 45% (90 questionnaires returned). The majority of the respondents (88%) were international visitors, mostly from the United Kingdom. The respondents were mostly executive or managerial (31%), and 19% were retired. Almost 80% of the visitors to Shamwari had a tertiary qualification. Forty-two percent of the respondents had previously visited other reserves in South Africa, mostly frequenting National Parks. Thirty-five percent of these respondents had previously visited private reserves in South Africa, dominated by the international respondents (43%), in comparison to 18% of South Africans. Forty percent of the respondents said that the most important criterion in selecting Shamwari was the variety of wildlife present, and that the availability of the “big five” species played an important role in their selection. More than half of the respondents (60%, n = 52) indicated that they would choose to visit a different reserve if their preferred species were not stocked at Shamwari. Of these respondents, few listed extralimital species such as the cheetah (8%, n = 7) or the giraffe (3%, n = 3) as their preferred animals to see.

According to the weighted scoring system, the lion was ranked by the respondents as the most preferred large mammal, followed by leopard, elephant, black wildebeest and the cheetah (table 1). The black wildebeest and cheetah were the only extralimital species ranked within the top five preferred large mammal species, but no significant difference was found in the weighted scores between indigenous and extralimital species (t = 2.16, *p* = 0.30, df = 13) (table 1).

**Table 1:** The most preferred large mammal species to see according to questionnaires issued to tourists at Shamwari Private Game Reserve, ranked according to weighted scores [24].

Species	Weighted score
**Lion**	4.04
**Leopard**	3.56
**Elephant**	3.22
**Black Wildebeest**	3.00
* **Cheetah**	2.97
**Warthog**	2.50
**Black Rhino**	2.38
* **Giraffe**	2.31
**Brown Hyena**	2.22
**Hippopotamus**	2.04
**Plains Zebra**	2.00
* **White Rhino**	1.82
**Kudu**	1.75
**Buffalo**	1.29
**Springbok**	1.00
**Impala**	1.00

*Extralimital species to Shamwari Private Game Reserve.

No significant difference was found in the average amount tourists were willing to pay to view indigenous versus extralimital species (t = 1.07, *p* = 0.31, df = 12). The largest monetary value was attached to the big cats, firstly leopard, followed by cheetah and lion.

**Figure 2:**
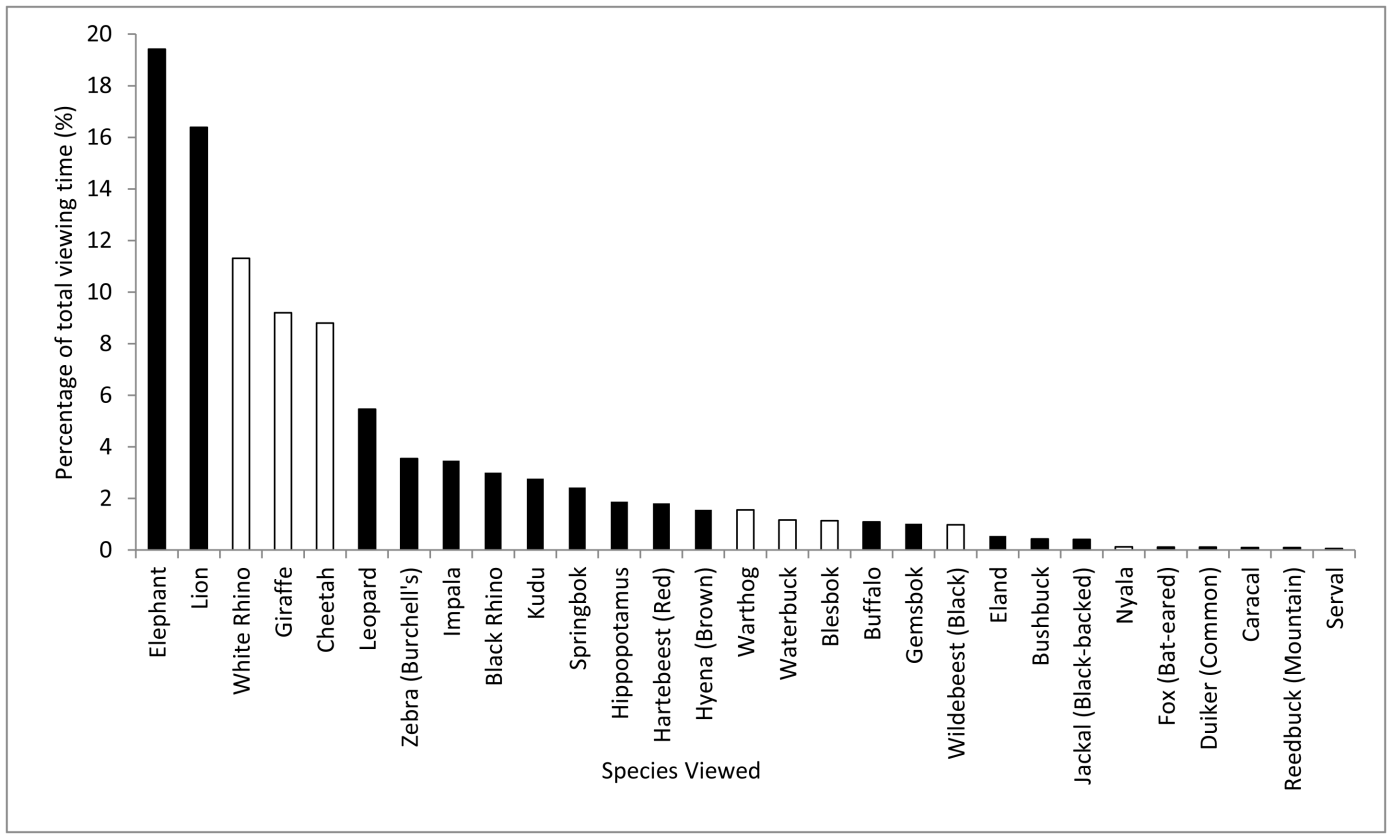
Proportion of total time spent viewing indigenous (black bars) and extralimital species (white bars) on game drives in Shamwari Private Game Reserve.

During 243 hours of game drives (n = 80, 14,597 minutes), 80% of the time was spent driving and 20% of the time was spent viewing mammals. Of the viewing time 19% was spent observing elephants, followed by 16% on lions, and declining to 0.1% for species such as caracal (*Caracal caracal*), mountain reedbuck (*Redunca fulvorufula*) and serval (*Felis serval*) (table 2, figure 2). No significant difference (t = −0.33, *p = *0.74, df = 27) was found in total time spent viewing indigenous versus extralimital species. However the majority (61%) of the time was spent viewing indigenous species, where on average 114.9 min (± SD 192.4) was spent viewing each species, compared to 39% that was spent viewing extralimital species (mean = 138.8 min ± SD 149.7) (figure 2).

**Table 2:** Total number of stops and total viewing time spent on each large mammal species in Shamwari Private Game Reserve, ranked according to viewing time.

Species	Total viewingtime (min)	Total numberof stops	Average time spent at eachstop (min)	Percentage of total viewing time (%)
**Elephant (** ***Loxodonta africana*** **)**	692.26	62	32.91	19.42
**Lion (** ***Panthera leo*** **)**	583.93	34	49.74	16.38
* **White Rhino (** ***Ceratotherium simum*** **)**	403.08	76	15.96	11.31
* **Giraffe (** ***Giraffa camelopardalis*** **)**	327.81	65	15.60	9.20
* **Cheetah (** ***Acinonyx jubatus*** **)**	313.51	26	36.97	8.80
**Leopard (** ***Panthera pardus*** **)**	194.82	17	41.80	5.47
**Plains Zebra (** ***Equus quagga*** **)**	126.55	61	6.22	3.55
* **Impala (** ***Aepyceros melampus*** **)**	123.59	79	4.72	3.47
**Black Rhino (** ***Diceros bicornis*** **)**	107.07	18	17.85	3.00
**Kudu (** ***Tragelaphus strepsiceros*** **)**	98.61	66	4.74	2.77
**Springbok (** ***Antidorcas marsupials*** **)**	86.44	43	6.21	2.43
**Hippopotamus (** ***Hippopotamus amphibious*** **)**	67.05	15	14.06	1.88
**Red Hartebeest (** ***Alcelaphus buselaphus*** **)**	64.45	40	4.73	1.81
**Brown Hyena (Parah** ***yaena brunnea*** **)**	55.73	15	11.42	1.56
* **Warthog (** ***Phacochoerus africanus)***	55.53	45	4.09	1.56
* **Waterbuck (** ***Kobus ellipsiprymnus*** **)**	41.42	21	5.72	1.16
* **Blesbok (** ***Damaliscus dorcas phillipsi*** **)**	40.72	22	5.62	1.14
**Buffalo (** ***Syncerus caffer*** **)**	38.84	4	9.71	1.09
* **Gemsbok (** ***Oryx gazella*** **)**	36.57	23	5.56	1.03
* **Black Wildebeest (** ***Connochaetes gnou*** **)**	34.78	24	3.77	0.98
**Eland (** ***Tragelaphus oryx*** **)**	19.44	11	5.74	0.55
**Bushbuck (** ***Tragelaphus scriptus*** **)**	15.47	21	2.20	0.43
**Black-backed Jackal (** ***Canis mesomelas*** **)**	14.66	8	4.54	0.41
**Nyala (** ***Tragelaphus angasii*** **)**	4.47	3	0.00	0.13
**Bat-eared Fox (** ***Otocyon megalotis*** **)**	4.12	3	4.07	0.12
**Common Duiker (** ***Sylvicapra grimmia*** **)**	4.08	6	1.83	0.11
**Caracal (** ***Caracal caracal*** **)**	3.49	2	0.00	0.10
**Mountain Reedbuck (** ***Redunca fulvorufula*** **)**	3.37	3	1.98	0.10
**Serval (** ***Felis serval*** **)**	2.16	1	2.16	0.06

*Extralimital species, species which historically did not occur there and have been introduced to Shamwari.

**Figure 3:**
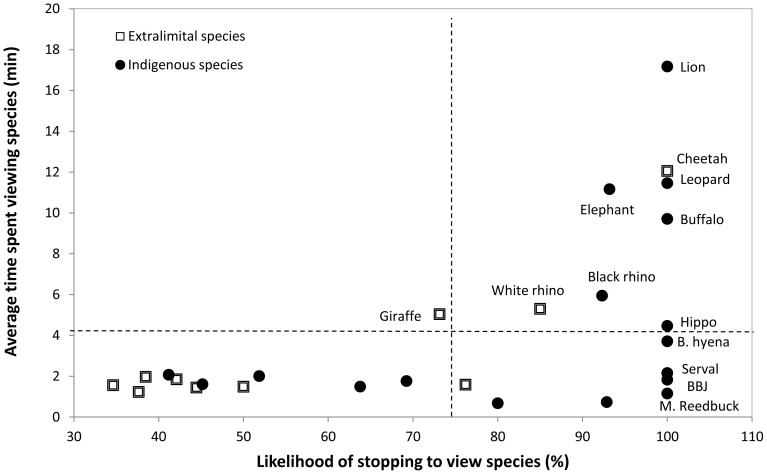
Relationship between the likelihood of stopping to view a species and average time spent viewing indigenous species on game drives at Shamwari Private Game Reserve. Average of all species indicated by dashed horizontal (time) and vertical (likelihood) lines.

No significant difference was found between the average time spent viewing indigenous versus extralimital species (t = 0.58, *p = *0.34, df = 27). However, when the total averages of each species were calculated, it was evident that more time (82.3 min) was spent viewing indigenous compared to extralimital species (33.6 min). Six indigenous species (lion, leopard, elephant, buffalo, black rhino and hippopotamus) and three extralimital species (cheetah, white rhino and giraffe) were viewed for longer than the average time calculated across all species (figure 3). For the members of the “big five” the likelihood of stopping to view these species was above 70% (figure 3). A significant relationship was found between the likelihood of stopping to view an animal and the average time spent viewing (F_1, 25_ = 9.83, *p*<0.05, R^2^ = 0.28) (figure 2). Nine species were always stopped for when they were sighted (figure 3). All of these, with the exception of the cheetah, are indigenous to Shamwari (figure 3). However, no significant relationship was found between the likelihood of stopping to view a species and whether the species was indigenous or not (Z = 1.45, *p = *0.15, df = 25).

Based on viewing-availability proportions, the five most important mammal species in terms of the Johnson’s method [32] were the serval, nyala, mountain reedbuck and black-backed jackal (table 3). A significant difference was found in the ranking between indigenous and extralimital species (Z = 2.80, *p*<0.05, df = 22), where the proportion of viewing indigenous species in terms of availability was higher than extralimital species (table 3).

**Table 3:** Relative importance rank of species based on proportions of viewing and the availability of large mammal species in Shamwari Private Game Reserve, based on the Johnson Method [32].

Species	Viewing vs. Availability(Johnson method) (and rank)	Tourist importance rank
**Serval (** ***Felis serval*** **)**	−26(1)	1
**Buffalo (** ***Syncerus caffer*** **)**	−24(2)	2
* **Nyala (** ***Tragelaphus angasii*** **)**	−22(3)	3
**Mountain Reedbuck (** ***Redunca fulvorufula*** **)**	−20(4)	4
**Black-backed Jackal (** ***Canis mesomelas*** **)**	−18(5)	5
**Common Duiker (** ***Sylvicapra grimmia*** **)**	−16(6)	6
**Leopard (** ***Panthera pardus*** **)**	−14(7)	7
**Hippopotamus (** ***Hippopotamus amphibious*** **)**	−11(8)	8
**Brown Hyena (** ***Parahyaena brunnea*** **)**	−11(8)	9
**Black Rhino (** ***Diceros bicornis*** **)**	−8(9)	10
* **Cheetah (** ***Acinonyx jubatus*** **)**	−4(10)	11
**Bushbuck (** ***Tragelaphus scriptus*** **)**	−4(10)	12
**Eland (** ***Tragelaphus oryx*** **)**	−4(10)	13
**Lion (** ***Panthera leo*** **)**	1(11)	14
* **Gemsbok (** ***Oryx gazella*** **)**	1(11)	15
* **Blesbok (** ***Damaliscus dorcas phillipsi*** **)**	5(12)	16
**Black Wildebeest (** ***Connochaetes gnou*** **)**	5(12)	17
* **Waterbuck (** ***Kobus ellipsiprymnus*** **)**	8(13)	18
**Elephant (** ***Loxodonta africana*** **)**	10(14)	19
**Springbok (** ***Antidorcas marsupialis*** **)**	12(15)	20
* **White Rhino (** ***Ceratotherium simum*** **)**	14(16)	21
* **Giraffe (** ***Giraffa camelopardalis*** **)**	17(17)	22
**Red Hartebeest (** ***Alcelaphus buselaphus*** **)**	17(18)	23
**Kudu (** ***Tragelaphus strepsiceros*** **)**	20(19)	24
* **Warthog (** ***Phacochoerus africanus*** **)**	22(20)	25
* **Impala (** ***Aepyceros melampus*** **)**	25(21)	26
**Plains Zebra (** ***Equus quagga*** **)**	25(21)	27

*Extralimital species, species which historically did not occur on Shamwari and have been introduced there.

## Discussion

We present a case study where we combine stated preference techniques with observational data to determine the ecotourism value of indigenous and extralimital species in PPAs. No significant difference was found between indigenous and extralimital species in the weighted scoring system, average amount tourists were WTP, viewing time or likelihood of stopping to view mammal species. This strongly suggests tourists do not have a specific preference for extralimital species in PPAs.

Of the 10 extralimital species stocked at Shamwari, the cheetah was the only extralimital species that scored highly as one of the top animals to see in both the questionnaire and in the time spent viewing. Other studies also highlighted tourists’ preference for cheetah [19], [20]. This charismatic species is often ranked with the leopard, lion, rhino and elephant as the most popular species among tourists [18].

During game drives, tourists were mostly attracted to elephants, followed by the large carnivores, i.e. lion, leopard and cheetah. With the exception of the cheetah, these species are all members of the “big five” and are indigenous to Shamwari. Lion, leopard, cheetah and elephant also scored the highest in terms of the average amount of time spent at every animal viewing. This concurs with previous studies, where these charismatic species were highly ranked as tourist attractions [30], [20]. In the Addo Elephant National Park (AENP), the majority of self-guided tourists listed elephants as an important reason for visiting the park [34]. In Tanzania, the lions attracted the most vehicles during a game drive, and 29% of viewing time was spent with these large carnivores [19].

The type of species encountered on a game drive may influence the amount of time spent at the sighting, however there are other variables that may influence tourist preferences. During game drives, the rare species or species that were less frequently encountered such as the serval, buffalo and nyala played an important role in attracting tourists’ attention. This is in accordance with previous studies where rare species were found to be more valuable than common species [35].

There is a difference between tourists’ stated preferences in the questionnaires and their observed preferences as measured on game drives. In particular, a higher value was placed on viewing the indigenous black rhino in questionnaires, whereas on game drives, a larger proportion of time was devoted to viewing the white rhino. White rhino are usually found in open habitats [36], increasing their visibility, as opposed to the shyer black rhino. Species availability and visibility may thereby influence the time allocated to different species during game viewing. The same was found with giraffe, which were frequently encountered during game drives and a large amount of time was spent at giraffe sightings, even though this species did not score highly as a popular species to see in the questionnaires. When located, however, a larger proportion of time was spent at black rhino sightings, compared to white rhino or giraffe, even though they were encountered less frequently during game drives.

We therefore suggest that species type is not the only determining factor, but there may be other variables that influence tourists’ preferences of large mammal species. The marketing of a reserve plays an important role in raising tourist expectations [37], [20]. Tourists’ preferences may also be influenced by their current knowledge of a species [15], [38]. Studies have found that visitors better appreciate the reserve they are visiting when they are taught about the natural and cultural values of the PA [39]. This suggests bringing more awareness to the indigenous species and educating tourists during game drives may increase tourist satisfaction in PPAs.

Our findings do not unequivocally support or reject the hypothesis that tourists having a preference for viewing extralimital species, and we can conclude that some extralimital species have value for ecotourism, but others not. Revealingly, among the herbivores, it is the larger species (e.g. giraffe, white rhino) that add value, and the smaller species (e.g. impala, nyala) are of lesser interest. This is important, as these larger species will occur at lower densities by virtue of their body size, and are also easier to manage. This is because they are easier to locate to monitor and also remove if needed. In contrast, the small herbivores, and hence less interesting species, such as impala and nyala may occur at relatively higher densities, and are more difficult to manage, as they are less easy to locate for monitoring and management actions such as removal. Thus, there is some alignment between the desirability of introducing alien herbivores and the risks they present in terms of management of their impacts. This issue needs to be further explored.

Without question, PAs are essential to the conservation of biodiversity [39] and PPAs play an important role in contributing to the PA estate. Stocking PPAs with high numbers of extralimital species however is not required to achieve tourist satisfaction. Certain extralimital species may contribute to the game viewing experience, however tourists do not have a specific preference for these species *per se*. Many other contributing factors play an important role in achieving tourist satisfaction. We thereby strongly recommend that management of PPAs should focus more on the principle goal of conserving biodiversity and rethink their current extralimital stocking rates.

## Supporting Information

Appendix S1 (PDF)
