# Supplementary material for: Understanding Tourists’ Preference for Mammal Species in Private Protected Areas: Is There a Case for Extralimital Species for Ecotourism?
Source: PLoS One. 2014 Feb 5;9(2):e88192. doi: 10.1371/journal.pone.0088192 (PMC3914921; doi:10.1371/journal.pone.0088192)
Supplement: Appendix S1 — (PDF) [file pone.0088192.s001.pdf]

Appendix

QUESTIONNAIRE

I am doing my PhD in Zoology at the Centre for African Conservation Ecology at the Nelson Mandela Metropolitan University where I am analysing the Value of Biodiversity for Ecotourism. As part of my study I am looking at Shamwari visitors' preference to different mammal species. Your opinion is thus of great value to my research and it would be greatly appreciated if you would take a few minutes to complete this simple **anonymous** questionnaire.

---

1. Country of Residence: ________________________
2. Occupation: ________________________
3. Gender:

Male	Female

4. Age Group:

Under 18	18 to 24	25 to 34	35 to 44	45 to 54	55 to 64	Over 65

5. Highest Qualification:

Primary School	High School	Tertiary Education

6. How did you find out about this particular reserve?

Magazine	Newspaper	Internet	Agent recommended	Friend recommended

Other (Please state): ___________________________

7. How long is your visit to this reserve:_______________________

8. Have you previously visited any other reserves in South Africa?

Yes	No

9. If yes, which reserves have you visited?

National Parks	Provincial Parks	Private Reserves

10. Shamwari Game Reserve is a Private Reserve. What made you choose **Shamwari**? Please rank in order of importance from 1 to 5, where 1 is very important and 5 is not that important.

Variety of Wildlife	
Pristine Landscapes	
Luxurious Accommodation	
Availability of the 'Big Five'	
Location of the Reserve	

11. Please rank your top **FIVE** answers to this next question: If you could only see one animal at this reserve, which one would be your first choice? Rank your favourite as “1”, the next favourite as “2” and so on. Only give us your top five choices.

A	Lion		L	Springbok	
B	Black Rhino		M	Impala	
C	White Rhino		N	Warthog	
D	Leopard		O	Gemsbok	
E	Cheetah		P	Red Hartebeest	
F	Buffalo		Q	Black Wildebeest	
G	Elephant		R	Blesbok	
H	Giraffe		S	Nyala	
I	Hippopotamus		T	Waterbuck	
j	Zebra		U	Bushbuck	
K	Kudu		V	Brown Hyena	

12. Of all the animals in the list above, list the THREE **least favourite/important** animals on your list of animals to see:

____

13. If your top FIVE choices were not available at this reserve, but was available at a reserve nearby, would you still come here or would you have gone to a reserve that does stock this animal?

I would still have come here		I would have looked for another reserve	

14. ‘Willingness to Pay’ is an economic method used to evaluate unpriced goods or services. If only ONE of each of your top FIVE animals were available, how much would you be ‘willing to pay’ to see this animal? (This information is purely for research purposes, and will not be used to solicit sales of any kind). Please tick a value.

Animal 1	$100	$200	$300	$400	$500
Animal 2	$100	$200	$300	$400	$500
Animal 3	$100	$200	$300	$400	$500
Animal 4	$100	$200	$300	$400	$500
Animal 5	$100	$200	$300	$400	$500

15. When viewing your preferred/favourite animal, please indicate how important the following features are to you on a scale of 1 to 5, where 1 is most important and 5 is not important at all.

Size of animal	1	2	3	4	5
Group size (e.g. Herd, pack size etc.)	1	2	3	4	5
Animal feeding (e.g. Herbivore grazing)	1	2	3	4	5
Animal hunting (e.g. Predator on a kill)	1	2	3	4	5
Presence of juveniles (calves, cubs etc)	1	2	3	4	5

Centre for African Conservation Ecology
ecological research for tomorrow

Thank you very much for your time
Kristine Maciejewski
krismacski@gmail.com

Nelson Mandela Metropolitan University
for tomorrow
